# Development of a Simplified Geriatric Score-4 (SGS-4) to Predict Outcomes After Allogeneic Hematopoietic Stem Cell Transplantation in Patients Aged over 50

**DOI:** 10.3390/cancers17203278

**Published:** 2025-10-10

**Authors:** Eugenia Accorsi Buttini, Alberto Zucchelli, Paolo Tura, Gianluca Bianco, Daniele Avenoso, Giovanni Campisi, Mirko Farina, Gabriele Magliano, Enrico Morello, Vera Radici, Nicola Polverelli, Domenico Russo, Alessandra Marengoni, Michele Malagola

**Affiliations:** 1 Department of Clinical and Experimental Sciences, University of Brescia, 25123 Brescia, Italy; p.tura@unibs.it (P.T.); vera.radici@unibs.it (V.R.); michele.malagola@unibs.it (M.M.); 2 Hematology Division, ASST Spedali Civili Brescia, 25123 Brescia, Italy; 3 Aging Research Center, Department of Neurobiology, Care Sciences and Society, Karolinska Institutet, 17177 Stockholm, Sweden; 4 Adult Bone Marrow Transplant Unit, ASST Spedali Civili of Brescia, Department of Clinical and Experimental Sciences, University of Brescia, 25123 Brescia, Italy; 5 Laboratorio Centrale di Analisi Chimico Cliniche, ASST Spedali Civili di Brescia, 25123 Brescia, Italy; 6 Unit of Bone Marrow Transplantation and Cellular Therapies, Division of Hematology, Fondazione Istituto di Ricovero e Cura a Carattere Scientifico Policlinico San Matteo, 27100 Pavia, Italy; n.polverelli@smatteo.pv.it

**Keywords:** allogeneic stem cell transplantation, comprehensive geriatric assessment, fitness, simplified geriatric score

## Abstract

**Simple Summary:**

Allogeneic stem cell transplantation represents a potentially curative option for several hematologic malignancies; however, assessing patient fitness, particularly in older adults, remains a clinical challenge. The Comprehensive Geriatric Assessment (CGA) is the gold standard for evaluating functional, cognitive, and physical domains, but its complexity limits routine application. In this prospective study, we developed and validated a Simplified Geriatric Score-4 (SGS-4) that includes only gait speed, hand grip strength, the Geriatric 8 questionnaire, and sex. Among patients aged 50 years or older undergoing transplantation, SGS-4 effectively stratified individuals into three fitness categories—fit, pre-frail, and frail—each associated with distinct survival and relapse outcomes. This simplified tool may facilitate the integration of geriatric assessment into everyday transplant practice, enabling more accurate risk stratification and personalized treatment planning for older patients.

**Abstract:**

**Background**: The Comprehensive Geriatric Assessment (CGA) has proven to be a valuable tool for providing a more comprehensive health evaluation of allogeneic stem cell transplantation (allo-SCT) recipients. **Methods**: We prospectively conducted a CGA in 135 consecutive patients aged ≥50 years who underwent allo-SCT between 2020 and 2023. Each CGA domain was individually analyzed for its association with overall survival (OS), non-relapse mortality (NRM), and cumulative incidence of relapse (CIR). Subsequently, a Simplified Geriatric Score-4 (SGS-4) was developed in a subset of 99 patients using two-factor analysis (FA) with oblimin rotation and Bartlett’s method applied to all CGA domains and sex. Based on the derived component weights, the SGS-4 formula was established as follows [Gait Speed] + 2 × [Hand Grip] + Geriatric 8 + 1.5 × [Sex]. ROC analysis defined three fitness groups, frail (≤13), prefrail (>13–22.5), and fit (>22.5). **Results**: Reduced hand grip strength and impaired mini mental state examination (MMSE) were associated with worse OS and higher NRM. Vulnerable Elders Survey (VES-13) and Fondazione Italiana Linfomi (FIL) scores also indicated poorer OS, though with uneven group sizes. Other CGA domains and the Hematopoietic Cell Transplantation–Comorbidity Index (HCT-CI) showed no significant prognostic value. The SGS-4 effectively stratified patients into three fitness groups, with those in the frail category experiencing lower OS and an increased risk of relapse. **Conclusions**: The new SGS-4 based on three CGA domains (gait speed, hand grip, Geriatric 8) and sex effectively predicts OS and CIR risk in patients aged ≥50 years undergoing allo-SCT. The study’s small sample size and disease heterogeneity warrant further validation in larger cohorts.

## 1. Introduction

Allogeneic stem cell transplantation (allo-SCT) represents the best-established curative treatment for many hematologic malignancies [1,2]. Due to the significant morbidity and mortality associated with transplant, this treatment approach was initially limited to patients younger than 45–50 years. In recent decades, the median age of the general population has progressively increased and, considering the high prevalence of hematological malignancies among older adults, the number of patients above the age of 60 potentially eligible for transplant has grown year by year [3,4]. Furthermore, the introduction of reduced-intensity and non-fully myeloablative preparative regimens, along with major advancements in donor selection, GVHD prophylaxis, and anti-infective therapies, has led to a growing number of older adults being referred for allo-SCT [5].

At present, advanced age does not represent an absolute threshold for allo-SCT, but the selection of eligible patients older than 50 years remains crucial to reduce the non-relapse mortality (NRM). The criteria currently used to evaluate potential risks of toxicity are based on comorbidity indices such as the Hematopoietic Cell Transplantation–Comorbidity Index (HCT-CI) and the Charlson Comorbidity Index [6,7,8]. Comorbidity alone, however, is insufficient to capture all the patient’s health domains, particularly the frailty [9,10]. The term frailty is used to describe an increased vulnerability resulting from cumulative deficits across multiple physiological systems, leading to diminished resilience to stressors such as cancer and its treatment, including allo-SCT. The Comprehensive Geriatric Assessment (CGA) is a multidimensional, interdisciplinary diagnostic process composed of various validated scores—each designed to assess a specific domain of health in older adults, such as physical function, nutrition, cognition, mood, social support, and functional independence. As such, CGA represents a fundamental tool for identifying and quantifying frailty in this population and defining the overall patient’s fitness. For this reason, we designed a prospective study to evaluate the predictive value of individual CGA components in determining outcomes among allo-SCT candidates aged over 50 years, and to develop a prognostic score, the simplified geriatric score-4 (SGS-4), based on the most reliable domains of the GCA assessment, in order to support clinical decision-making without imposing a significant burden on routine practice.

## 2. Materials and Methods

### 2.1. Patient Population and Geriatric Assessment

In January 2020, our center started a frailty prospective assessment program in all adult patients aged 50 years or older scheduled to receive allo-SCT at our institution. The frailty assessment involved a CGA evaluation by a Geriatrician across domains of functional status, physical performance, cognitive function, and comorbidities, with a median completion time of 30–45 min. Specifically, the CGA included activities of daily living (ADL) [11], instrumental activities of daily living (IADL) [12], Geriatric 8 (G8) [13], Vulnerable Elders Survey (VES-13) score [14], clinical frailty score (CFS) [15], a gait speed test [16], chair stand and hand grip tests [17], mini-cognitive assessment instrument (mini-cog) [18], mini mental state examination (MMSE) [19], HCT-CI [6] and Fondazione Italiana Linfomi (FIL) score [20]. In the FIL score, patients were classified as fit if they met all the following criteria: an ADL score of 6, an IADL score of 8, no grade 3–4 comorbidities, and fewer than five grade 2 comorbidities according to the CIRS scale. The Supplementary Material reports additional information on the thresholds used for the various scores and measurement indices.

The CGA was performed within a 2- to 6-week window prior to the initiation of allo-SCT conditioning. By the end of December 2023, a total of 135 consecutive patients aged ≥50 years had been evaluated. Using the collected data, we first assessed the individual impact of each CGA component on overall survival (OS), NRM, and cumulative incidence of relapse (CIR).

Given that the CGA requires a trained specialist and considerable time—factors that limit its feasibility in routine clinical practice—this study focused on developing a simplified score, the SGS-4, composed of only three geriatric scales plus sex, designed to predict outcomes without adding a significant time burden.

The study was approved by the local Ethical Committee and conducted in compliance with current National and European legislation on clinical trials, in accordance with the Declaration of Helsinki and the principles of Good Clinical Practice. The study was approved by the Ethical Committee of ASST Spedali Civili di Brescia, protocol code NP5593. All the patients signed the informed consent form for registration and data collection.

### 2.2. Statistical Analysis and Mathematical Model

Categorical data were presented as numbers and percentages, and continuous data as median and range. GCA impairments were identified using established cut-off points, as outlined in Table S1 of the Supplementary Material.

The OS was estimated using the Kaplan–Meier method. The NRM and the CIR were analyzed using the cumulative incidence of competing events with the Fine–Gray method.

A multi-step approach was used to develop the score. First, the set of variables to be potentially included was identified: all variables from the CGA and sex were considered. Second, the presence of latent “factors” within the dataset was investigated using factor analysis (FA). FA is a statistical technique that models the shared variance among a set of observed variables to identify a smaller number of unobserved (latent) variables that account for the observed correlation structure. The variability of each observed variable is decomposed into common variance (shared with other variables and explained by latent factors) and unique variance (specific to that variable, including measurement error). This approach was selected for score development because it reduces the risk of overfitting, improves generalizability, and is particularly suited to datasets with limited sample sizes. A heterogeneous correlation matrix was used to account for the mixed nature of the variables (categorical and continuous). A scree-plot was used to identify the optimal number of factors to retain in the analysis, using the “elbow method”. A two-factor FA was then performed, using oblimin rotation, as partial correlation among latent factors was expected in geriatric constructs (e.g., physical performance and global health). Bartlett’s method for factor score estimation was applied to minimize the sum of squared unique variances. Variables with absolute factor loadings ≥ 0.3 were considered relevant for further analysis. Based on the clinical expertise of two geriatricians (A.Z and G.B), the factor including gait speed, hand grip strength, G8 score, and sex was selected as a meaningful latent construct representing overall fitness. In the third step, the continuous variables (hand grip strength and gait speed) were divided into quartiles and combined with the categorical variables (G8 and sex) to build a linear formula. Each variable was weighted proportionally to its factor loading derived from FA. The final formula was as follows:

Score = [Gait Speed] + 2 × [Handgrip Strength] + [G8] + 1.5 × [Sex].

For the application of this formula, the values to be entered must be those specified in Table 1.

Lastly, Receiver Operating Characteristic (ROC) analysis was applied to determine two cut-offs for the score: the first cut-off maximized specificity while preserving a sensitivity ≥ 0.9, whereas the second maximized sensitivity while retaining specificity ≥ 0.9. Based on these thresholds, participants were classified into three fitness categories, frail (*n* = 13; score ≤ 13), prefrail (*n* = 62; score > 13 to ≤ 22.5), and fit (*n* = 24; score > 22.5). All analyses were conducted with R 4.3.6 (R Foundation for statistical computing—Vienna; Austria).

## 3. Results

### 3.1. Patients and Transplant Characteristics

Table 2: Clinical and transplant characteristics of the patients evaluated before allo-SCT.

A total of 135 patients completed the CGA and underwent allo-SCT. The median age at transplant was 63 years (range 50–75), with 63.7% (86/135) being male. Acute myeloid leukemia (AML) was the most common indication (39.3%). According to the CIBMTR-based Disease Risk Index (DRI), 3 patients were low risk, 95 intermediate, 35 high, and 2 very high risk. The Karnofsky Performance Status (KPS) was ≥90 in 93.4% of cases. HCT-CI scores were 0 in 20%, 1–2 in 27.4%, and ≥3 in 52.6% of patients. Based on the FIL score, 97.8% (132/135) were classified as “fit.” At transplant, 39% were in first complete remission. Donor types included related (24%), matched unrelated (47%), and haploidentical (29%). Peripheral blood stem cells (PBSCs) were used in 92% of cases. Conditioning regimens were myeloablative (MAC) in 63% and reduced-intensity (RIC) in 37%. GVHD prophylaxis included CyA + MMF (2.2%), CyA + MTX (3%), CyA + MTX + ATG (57.8%), and post-transplant cyclophosphamide (PTCy), 20%.

### 3.2. Overall Survival (OS), Non-Relapse Mortality (NRM) and Cumulative Incidence of Relapse (CIR)

**Figure 1:**
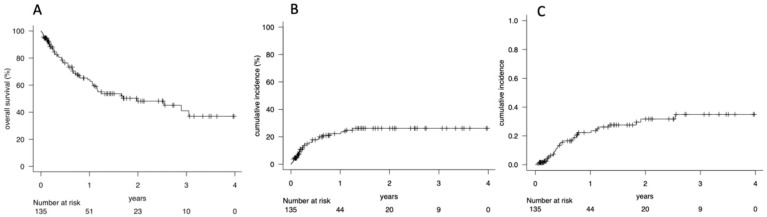
(**A**) Overall survival (OS), (**B**) non-relapse mortality (NRM), and (**C**) cumulative incidence of relapse (CIR) of 135 patients submitted to GCA and to allo-SCT. OS at 1, 2, and 4 years: 63% (95% CI 51–71%), 48% (95% CI 37–57%), and 37% (95% CI 23–50%) (**A**) NRM at 1, 2, 4 years: 25% (95% CI 16–32%), 26% (95% CI 18–35%), and 26% (95% CI 51–71%) (**B**) CIR at 1, 2, 4 years: 24% (95% CI 17–33%), 32% (95% CI 22–42%), and 35% (95% CI 24–46%) (**C**).

After a median follow up of 7.5 months (range 0.01–47.8), the OS at 1, 2, and 4 years is 63% (95% CI 51–71%), 48% (95% CI 37–57%), and 37% (95% CI 23–50%), respectively (Figure 1A). The 1, 2, 4-year NRM is 25% (95% CI 16–32%), 26% (95% CI 18–35%), and 26% (95% CI 51–71%), respectively (Figure 1B). Among NRM causes, acute GVHD (aGVHD) occurred in 55 patients (41%), of whom 37 had grade II-IV disease. Chronic GVHD (cGVHD) developed in 15 patients (11%), with three cases classified as severe. The CIR at 1, 2, and 4 years is 24% (95% CI 17–33%), 32% (95% CI 22–42%), and 35% (95% CI 24–46%) (Figure 1C).

### 3.3. Comprehensive Geriatric Assessment and Outcomes

Table 3 presents the analysis of OS, NRM, and CIR at 1 and 2 years, stratified by various GCA parameters in patients undergoing allo-SCT. Among disability domains, both the VES-13 and FIL scores were significantly associated with outcomes. Patients with a VES-13 score > 3 had a 1-year OS of 11% and a 1-year CIR of 58%, compared to 67% and 18%, respectively, in patients with a score ≤ 3 (*p* = 0.04 and *p* = 0.01). Similarly, patients classified as unfit according to the FIL score had a 1-year OS of 0% and a 1-year CIR of 100%, compared to 65% and 21% in fit patients (both *p* < 0.01). In the physical domain, patients with reduced hand grip strength had a significantly lower 1-year OS (45% vs. 68%, *p* < 0.01) and a higher 1-year NRM (34% vs. 21%, *p* = 0.05) compared to those with normal strength. Regarding cognitive function, patients with MMSE impairments had a significantly lower 1-year OS compared to those without impairments (19% vs. 66%; *p* = 0.03). No statistically significant differences in OS, NRM, or CIR were observed across the other geriatric assessment domains between patients with physical or cognitive impairments and those without.

### 3.4. Simplified Geriatric Score-4 (SGS-4) Design

**Figure 2:**
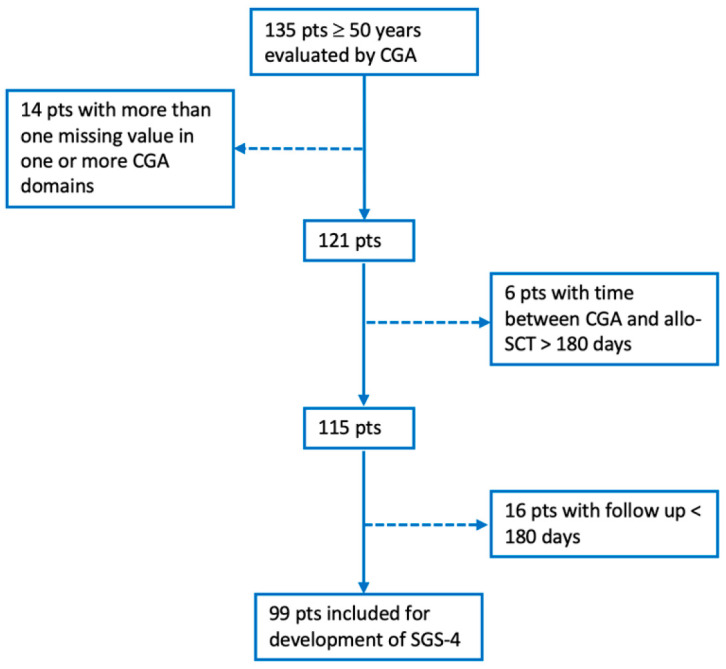
Flowchart for the selection of patients included in the factor analysis (FA). Table: GCA—comprehensive geriatric assessment; allo-SCT—allogeneic-stem cell transplantation; SGS-4—simplified geriatric score-4.

Figure 2 represents the flowchart for the selection of patients in whom we applied the FA method to identify the simplified score (SGS-4) for fitness assessment before allo-SCT. Out of the initial 135 patients who underwent CGA, 14 were excluded due to missing data in one or more CGA domains, 6 because the interval between CGA and allo-SCT exceeded 180 days, and 16 due to a follow-up period of less than 180 days. As a result, 99 patients were included in the final cohort for the development of SGS-4.

As previously described, the SGS-4 incorporated gait speed, hand grip strength, G8 score, and sex. It stratified patients into three fitness categories, frail (*n* = 13; score ≤ 13), prefrail (*n* = 62; score > 13 and ≤22.5), and fit (*n* = 24; score > 22.5). The frail group included patients with the poorest physical and mental condition, while the fit group represented those with the best performance. Baseline patient characteristics were well balanced across the three different fitness groups, with no statistically significant differences observed in age, underlying diagnosis, DRI, state of disease at allo-SCT, number of prior therapy lines, stem cell source, conditioning intensity, donor age, donor type, time to allo-SCT or incidence of acute and chronic GVHD (Table 4). This suggests that the fitness stratification was not confounded by these key clinical variables.

**Figure 3:**
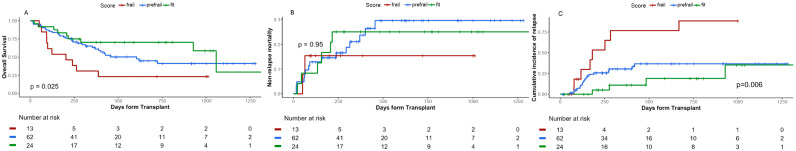
Overall survival (OS), non-relapse mortality (NRM), and cumulative incidence of relapse (CIR) of 99 patients submitted to allo-SCT according to the simplified risk score. OS at 1, 2, and 3.5 years were 30%, 24.5%, and 24.5% in the frail group; 62.7%, 40.8%, and 40.8% in the prefrail group; and 70.3%, 70.3%, and 29.3% in the fit group (**A**) NRM rates at 1, 2, and 3.5 years were 16.1%, 16.1%, and 16.1% in the frail group; 20.6%, 29.9%, and 29.9% in the prefrail group; and 25.0%, 25.0%, and 25.0% in the fit group (**B**) CIR at 1, 2, and 3.5 years were 69.2%, 80.4%, and 80.4% in the frail group; 30.4%, 36.4%, and 36.4% in the prefrail group; and 10.9%, 19.0%, and 35.3% in the fit group (**C**).

One-, two-, and three-year OS rates were 32.7%, 24.5%, and 24.5% in the frail group; 62.7%, 40.8%, and 40.8% in the prefrail group; and 70.3%, 70.3%, and 29.3% in the fit group (*p* = 0.025; Figure 3A). Corresponding NRM rates at 1, 2, and 3 years were 16.1%, 16.1%, and 16.1% in the frail group; 20.6%, 29.9%, and 29.9% in the prefrail group; and 25.0%, 25.0%, and 25.0% in the fit group (*p* = 0.95; Figure 3B). CIR at 1, 2, and 3 years were 69.2%, 80.4%, and 80.4% in the frail group; 30.4%, 36.4%, and 36.4% in the prefrail group; and 10.9%, 19.0%, and 35.3% in the fit group (*p* < 0.01) (Figure 3C).

## 4. Discussion

Patient fitness has emerged as a key determinant of outcomes, particularly in older individuals undergoing allo-SCT, but its systematic evaluation in routine clinical practice remains limited and not yet standardized [21]. Muffly and colleagues were the first to implement the CGA in allo-SCT candidates aged 50 years and older [22,23]. They demonstrated that impairments in self-care, poor mental health, difficulty walking, elevated serum C-reactive protein levels, and high comorbidity burden were all significantly associated with inferior survival after allo-SCT.

These findings have been independently validated by multiple groups, who also identified additional prognostically significant domains, including cognition, medication use, and frailty scales [24,25,26,27,28]. Given the increasing age of transplant candidates, there is a pressing need for reliable and time-efficient tools to support risk stratification for clinical decision-making [29]. In this study, we analyzed the impact of fitness on post-transplant outcomes and developed a simplified score (SGS-4) for prognosis prediction.

Our cohort included a high proportion of patients over 60 years of age, with more than half presenting a significant burden of comorbidities (HCT-CI ≥ 3), consistent with the findings from Italian and American registry studies [3,5]. Thus, this population is particularly suited to fitness evaluation. We assessed patients’ fitness, guided by geriatric expertise, using several scoring tools that cover physical and cognitive functions, disabilities, and comorbidities. The aim was to identify which factors were mostly associated with post-transplant outcomes.

As reported in Table 3, among physical performance measures, hand grip strength emerged as the strongest predictor of clinical outcome. Reduced muscle strength was significantly associated with worse OS (*p* < 0.01) and NRM (*p* = 0.05), highlighting the prognostic relevance of sarcopenia in hematologic malignancies, as previously observed in solid tumors [30,31]. In the cognitive domain, lower MMSE scores correlated with inferior OS (*p* = 0.03). This finding is known in the field of solid tumors, where it has been demonstrated that the presence of major neurocognitive disorder has direct prognostic value, independently of other geriatric factors, cancer type, and treatment strategy in older patients [32]. Moreover, both the VES-13 and the FIL score were predictive of OS (*p* = 0.04 and *p* < 0.01, respectively) and CIR (*p* = 0.01 and *p* < 0.01, respectively). VES-13 has previously shown strong associations with poor OS in solid tumor patients [33], while the FIL score was originally designed to support treatment decisions in elderly patients with diffuse large B-cell lymphoma and was proven to be helpful in identifying patients likely to benefit from curative-intent therapies [20,34]. Interestingly, in our series, traditional indices such as HCT-CI did not significantly correlate with OS or NRM, suggesting that comorbidity scores alone may inadequately reflect transplant-related risks in older patients.

Considering the results of the performance of each single tool on prognosis prediction, it appears clear that the challenge is to establish how to “pick the winner”. Moreover, if we focus on the significant tools that we have identified as significantly associated with outcome, it appears evident that the distribution of the patients between the fit and frail/prefrail group is unbalanced, reflecting the patients’ pre-transplant selection. To address these issues and refine our approach, we applied FA to the CGA domains and developed a simplified, fast, and reproducible predictive score for allo-HCT outcomes. This score, named SGS-4, incorporates gait speed, hand grip strength, G8, and sex.

The SGS-4 stratified patients into three fitness groups with distinct differences in OS and CIR. The frail group, which included patients with poor performance status, exhibited significantly worse survival and higher relapse rates. Specifically, the 2-year OS was 70.3% in the fit group versus 24.5% in the frail group (*p* = 0.025), while the 2-year CIR was 19% in the fit group compared to 80.4% in the frail group (*p* < 0.01) (Figure 3A,C). Notably, and apparently quite unexpectedly, the NRM seems not to be affected by patients’ fitness (Figure 3B).

This association between fitness and relapse risk is consistent with findings from Muffly et al. [23], who reported that slower walking speed was linked to higher relapse rates, and with a more recent study by Sung et al. [35]. In their work, Sung and colleagues evaluated the association of Fried’s Frailty Phenotype (FFP)—a geriatric assessment incorporating gait speed, grip strength, physical activity, exhaustion, and weight loss—with post-allo-SCT outcomes in 280 recipients aged over 60 years. The 2-year CIR was 21.4% in fit patients, 34.1% in prefrail patients, and 40% in frail patients, with a statistically significant difference between fit and prefrail groups (*p* = 0.044). Similarly, in solid oncology, frailty has been independently associated with breast cancer recurrence in older women [36].

Importantly, in our study, baseline clinical characteristics—including diagnosis, number of prior treatment lines, conditioning intensity, donor type and age, time from diagnosis to transplant, and incidence of GVHD—were evenly distributed across the three fitness groups (Table 4) and thus did not influence relapse risk. This supports the hypothesis that our score captures an underlying physiological reserve, not reflected by the standard risk stratification. Geriatric vulnerability may influence disease control, potentially through an attenuated graft-versus-leukemia (GVL) effect in frail patients. From this perspective, reduced muscular strength and sarcopenia (as captured by the hand grip and the gait speed tests) may correlate with a non-effective graft-versus-leukemia reaction and minimal residual disease control.

Moreover, emerging preclinical and clinical evidence supports this link between fitness and relapse risk. Factors such as malnutrition [37], gut microbiota disruption [38], and heightened systemic inflammation [39,40]—all common in frail populations—may favor disease recurrence.

Interestingly, while fitness significantly influenced OS and CIR, it did not impact NRM across the three groups. This may be attributed to the improvement of the transplant platform. In recent years, there has been a shift from standard myeloablative conditioning (MAC) to reduced-toxicity regimens, and the supportive care has significantly improved, contributing to reduced NRM.

The main strengths of our study include its prospective design, inclusion of a real-world population, and the statistical rigor of the factor analysis, which minimizes overfitting. Nevertheless, certain limitations should be acknowledged. The relatively small sample size and disease heterogeneity may limit the generalizability of our findings. Moreover, although our score is based on simple, easily obtainable parameters, its clinical utility should be validated in larger, multicenter studies and across different transplant platforms, possibly including a homogeneous cohort of patients (e.g., AML), and eventually integrating other factors, such as biochemical markers. We are currently planning such a multicentric study within the Centers accredited to the Gruppo Italiano Trapianto di Midollo Osseo (GITMO). Additionally, longer follow-up is required to confirm these findings.

In conclusion, while individual fitness domains may not sufficiently predict post-transplant survival on their own, their integration into a composite score significantly improves risk stratification in older adults. Looking ahead, routine incorporation of geriatric screening into transplant decision-making may help in identifying patients who could benefit from prehabilitation or modified transplant strategies. Supporting this, Salas et al. recently showed that frailty is a dynamic and potentially reversible condition that can be the target of appropriate pre-transplant interventions [41]. These findings open the door to prehabilitation programs to further improve patients’ outcomes.

## 5. Conclusions

The present study shows that the SGS-4 score, integrating gait speed, hand grip strength, G8, and sex, offers a simple and reproducible tool for fitness assessment and prognostic stratification in older allo-SCT recipients. Unlike conventional comorbidity indices, SGS-4 captures dimensions of physiological reserve that strongly influence overall survival and relapse risk. Notably, while patient fitness impacted survival and relapse, non-relapse mortality appeared unaffected, likely reflecting advances in transplant platforms and supportive care. These findings support the systematic use of geriatric-informed tools in clinical decision-making and suggest that frailty may represent a modifiable risk factor amenable to prehabilitation strategies. However, the relatively small cohort, disease heterogeneity, and limited follow-up represent important limitations. Larger, multicenter studies are warranted to validate SGS-4 and explore its integration with biological and molecular markers to further refine risk prediction.

## Figures and Tables

**Table 1:** Values to be used in the formula and the corresponding cut-off points for gait speed and hand grip strength and sex.

Values	Gait Speed (m/s)	Hand Grip (kg)	Sex
1	≤1.04	≤24.5	M
2	1.04 < X ≤ 1.17	24.5 < X ≤ 32.7	F
3	1.17 < X ≤ 1.29	32.7 < X ≤ 41.3	
4	1.29 < X ≤ 1.70	41.3 < X ≤ 57.6	

**Table 2:** Clinical and transplant characteristics of the 135 patients submitted to GCA.

Characteristics	N (%)
Number	135 (100)
Age at allo-SCT, years, median (range)	63 (50–75)
Male, n (%)	86 (63.7)
Diagnosis, n (%)	
AML	53 (39.3)
MDS	17 (12.6)
ALL	11 (8.1)
Lymphoma	13 (9.6)
MF	25 (18.5)
Other	16 (11.9)
DRI, n (%)	
Low	3 (2.2)
Intermediate	95 (70.4)
High	35 (25.9)
Very high	2 (1.5)
KPS, n (%)	
90–100	126 (93.4)
70–80	9 (6.6)
HCT-CI, n (%)	
0	27 (20)
1–2	37 (27.4)
≥3	71 (52.6)
FIL score, n (%)	
Fit	132 (97.8)
Unfit	3 (2.2)
Disease status, n (%)	
1st CR	53 (39.3)
2nd or subsequent CR	82 (60.7)
Donor type, n (%)	
Related	33 (24.4)
Unrelated	63 (46.7)
Haploidentical	39 (28.9)
Stem cell source, n(%)	
PBSC	124 (91.9)
BM	11 (8.1)
Conditioning regimen, n (%)	
MAC	85 (63)
RIC	50 (37)
GVHD prophylaxis, n (%)	
CyA + MFF	3 (2.2)
CyA + MTX	4 (3)
CyA + MTX + ATG	78 (57.8)
PTCy + Other	27 (20)
Other	23 (17)
Follow-up, months, median (range)	7.5 (0.01–47.8)

Table legend: n—number; Allo-SCT—allogeneic stem cell transplantation; AML—acute myeloid leukemia; MDS—myelodysplastic syndrome; ALL—acute lymphoblastic leukemia; MF—myelofibrosis; DRI—Disease Risk Index; KPS—Karnofsky Performance Status; HCT-CI—Hematopoietic Cell Transplantation–Comorbidity Index; FIL—Fondazione Italiana Linfomi; CR—complete remission; PBSCs—peripheral blood stem cells; BM—bone marrow; MAC—myeloablative conditioning; RIC—reduced-intensity conditioning; CyA—cyclosporine A; MMF—mycophenolate mofetil; MTX—methotrexate; ATG—antithymocyte globulin; PTCy—post-transplant cyclophosphamide; aGVHD—acute graft-versus-host disease; cGVHD—chronic graft-versus-host disease.

**Table 3:** Overall survival (OS), non-relapse mortality (NRM), and cumulative incidence of relapse (CIR) of 135 patients submitted to allo-SCT according to each geriatric assessment domain.

	Pts Evaluated	OS 1 y	OS 2 y	*p*	NRM 1y	NRM 2 y	*p*	CIR 1 y	CIR 2 y	*p*
ADL										
Any limitations	4	50	-		25	-		25	-	
No limitations	130	63	49	0.5	24	26	0.95	23	30	0.73
IADL										
Any limitations	118	30	50		46	24		35	35	
No limitations	15	66	30	0.11	21	46	0.11	22	21	0.72
G8, n										
≤13	51	52	46		24	24		31	37	
>13	83	67	48	0.7	24	29	0.9	17	26	0.23
VES 13, n										
≤3	14	11	11		30	30		58	58	
>3	120	67	52	0.04	23	26	0.93	18	28	0.01
FIL score										
FIT	132	65	50		24	27		21	30	
UNFIT	3	0	-	<0.01	0	23	0.41	100	-	<0.01
CFS										
FIT	75	63	55		23	23		22	35	
UNFIT	12	45	-	0.6	28	-	0.9	27	-	0.8
Gait Speed, m/s										
≤1.1	80	73	60		20	22		25	31	
>1.1	49	41	27	0.06	31	34	0.31	40	40	0.2
Chair Stand, times										
<14	90	65	50		21	22		25	32	
≥14	41	54	40	0.5	27	34	0.37	24	31	0.83
Hand Grip, Kg										
<26	37	45	30		34	38		28	41	
≥26	97	68	54	<0.01	21	21	0.05	20	28	0.6
Mini-Cog, n										
1–2	56	64	58		21	21		19	29	
3	77	60	39	0.28	24	29	0.44	26	31	0.45
MMSE, n										
≤24	13	19	19		52	52		29	48	
>24	121	66	50	0.03	22	24	0.2	22	29	0.56
HCT-CI, n										
0	27	88	33		17	17		18	25	
1–2	37	70	49		18	18		22	38	
≥3	71	55	37	0.2	29	34	0.2	28	30	0.88

Table legend: ADL—Activities of Daily Living; IADL—Instrumental Activities of Daily Living; G8—Geriatric 8; VES-13—Vulnerable Elders Survey-13; FIL—Fondazione Italiana Linfomi; CFS—clinical frailty score; Mini-Cog—mini-cognitive assessment instrument; MMSE—mini mental state examination; HCT-CI—Hematopoietic Cell Transplantation–Comorbidity Index.

**Table 4:** Distribution of patient characteristics across the three risk classes (frail, prefrail, and fit) of the simplified geriatric score-4 (SGS-4).

Characteristics	Total *N* = 99	Frail *N* = 13	Prefrail *N* = 62	Fit *N* = 24	*p*-Value
Age, years (range)	63.0 (56.0, 66.0)	64.0 (56.0, 68.0)	62.0 (57.0, 66.0)	63.0 (56.0, 65.0)	>0.9
Diagnosis, *n* (%)					
AL	41 (41.4)	10 (76.9)	23 (37.1)	8 (33.3)	0.10
MDS	11 (11.1)	2 (15.4)	6 (9.7)	3 (12.5)	
Lymphoma	14 (14.1)	0 (0)	11 (17.7)	3 (12.5)	
MPN	1 (1.0)	0 (0)	0 (0)	1 (4.2)	
MM	5 (5.1)	0 (0)	5 (8.1)	0 (0)	
Other	27 (27.3)	1 (7.7)	17 (27.4)	9 (37.5)	
DRI, *n* (%)					
1	2 (2.0)	0 (0)	1 (1.6)	1 (4.2)	0.08
2	70 (70.7)	7 (53.8)	50 (80.7)	13 (54.2)	
3	24 (24.3)	6 (46.2)	10 (16.1)	8 (33.2)	
4	2 (2.0)	0 (0)	1 (1.6)	1 (4.2)	
missing	1 (1)	0	0	1 (4.2)	
KPS, *n* (%)					
90–100	92 (92.9)	11 (84.6)	57 (91.9)	24 (100)	0.6
70–80	7 (7.1)	2 (15.4)	5 (8.1)	0 (0)	
HCT-CI, *n* (%)					
0	21 (21.2)	1 (7.7)	12 (19.4)	8 (33.3)	0.13
1–2	28 (28.3)	3 (23.1)	16 (25.8)	9 (37.5)	
≥3	50 (50.5)	9 (69.2)	34 (54.8)	7 (29.2)	
Disease status, *n* (%)					
1st CR	33 (33.3)	6 (46.2)	20 (32.3)	7 (29.2)	0.6
2nd or subsequent CR	66 (66.7)	7 (53.8)	42 (67.7)	17 (70.8)	
Line of Therapy, *n* (%)					
1	48 (49.5)	8 (61.5)	28 (45.2)	12 (52.0)	0.5
≥2	50 (50.5)	5 (38.5)	34 (54.8)	11 (48.0)	
Missing	1 (1.0)	0	0	1	
Stem cell source, *n* (%)					
PBSC	91(92.0)	12 (92.3)	56 (90.3)	23 (95.8)	0.9
BM	8 (8.0)	1 (7.7)	6 (9.7)	1 (4.2)	
Conditioning regimen, *n* (%)					
MAC	40 (40.4)	4 (30.7)	25 (40.3)	11(45.8)	0.7
RIC	59 (59.6)	9 (69.3)	37 (59.7)	13 (54.2)	
TCI score, *n*	2.5	2.8	2.7	3.0	0.4
Donor age, years (range)	37 (28–55)	40 (28–45)	36 (28–47)	38 (28–55)	0.9
Donor, *n* (%)					
Related	23 (23.2)	3 (23.1)	13 (21.0)	7 (29.2)	0.9
Unrelated	44 (44.5)	6 (46.1)	27 (43.5)	11 (45.8)	
Haploidentical	32 (32.3)	4 (30.8)	22 (35.5)	6 (25.0)	
Time to allo-SCT, months (range)	11.3 (5.7–54.9)	6.3 (5.7–10.2)	12.4 (7.1–29.0)	19.5 (6.7–54.9)	0.03
aGVHD	48 (48.5)	4 (30.8)	29 (46.8)	15 (62.5)	0.17
cGVHD	16 (16.2)	0 (0.0)	10 (16.1)	6 (25.0)	0.12

Table legend: *n*, number; AL—acute leukemia; MDS—myelodysplastic syndrome; MPN—Myeloproliferative Neoplasms; Multiple Myeloma—MM; DRI—Disease Risk Index; KPS—Karnofsky Performance Status; HCT-CI—Hematopoietic Cell Transplantation–Comorbidity Index; CR—complete remission; PBSCs—peripheral blood stem cells; BM—bone marrow; MAC—myeloablative conditioning; RIC—reduced-intensity conditioning; TCI—transplant conditioning intensity; allo-SCT—allogeneic stem cell transplantation; aGVHD—acute graft-versus-host disease; cGVHD—chronic graft-versus-host disease.

## Data Availability

Data are available from the corresponding author upon request.

## Supplementary Materials

The following supporting information can be downloaded at https://www.mdpi.com/article/10.3390/cancers17203278/s1; Table S1: Cut-off points of GCA domains.

## Abbreviations

The following abbreviations are used in this manuscript:

ADL	Activities of daily Living
aGVHD	acute Graft-versus-Host Disease
cGVHD	chronic Graft-versus-Host Disease
Allo-HCT	Allogeneic Hematopoietic Cell Transplantation
AML	Acute Myeloid Leukemia
ATG	Anti-T Lymphocyte Globulin
CGA	Comprehensive Geriatric Assessment
CIBMTR	Center for International Blood and Marrow Transplant Research
CSF	Clinical Frailty Scale
CIR	Cumulative Incidence of Relapse
CyA	Cyclosporine A
DRI	Disease Risk Index
FA	Factor Analysis
FIL	Fondazione Italiana Linfomi
G8	Geriatric 8 Screening Tool
GITMO	Gruppo Italiano Trapianto di Midollo Osseo
GVHD	Graft-versus-Host Disease
GVL	Graft-versus-leukemia
HCT-CI	Hematopoietic Cell Transplantation–Comorbidity Index
IADL	Instrumental Activities of daily Living
KPS	Karnofsky Performance Status
MAC	Myeloablative Conditioning
MMF	Mycophenolate Mofetil
MMSE	Mini Mental State examination
MTX	Methotrexate
MUD	Matched Unrelated Donor
NRM	Non-Relapse Mortality
OS	Overall Survival
PBSC	Peripheral Blood Stem Cells
PTCy	Post-Transplant Cyclophosphamide
RIC	Reduced-Intensity Conditioning
SGS-4	Simplified Geriatric Score (4 variables)
VES-13	Vulnerable Elders Survey-13
