# Correction: Accorsi Buttini et al. Development of a Simplified Geriatric Score-4 (SGS-4) to Predict Outcomes After Allogeneic Hematopoietic Stem Cell Transplantation in Patients Aged over 50. *Cancers* 2025, *17*, 3278

**DOI:** 10.3390/cancers18121913

**Published:** 2026-06-12

**Authors:** Eugenia Accorsi Buttini, Alberto Zucchelli, Paolo Tura, Gianluca Bianco, Daniele Avenoso, Giovanni Campisi, Mirko Farina, Gabriele Magliano, Enrico Morello, Vera Radici, Nicola Polverelli, Domenico Russo, Alessandra Marengoni, Michele Malagola

**Affiliations:** 1 Department of Clinical and Experimental Sciences, University of Brescia, 25123 Brescia, Italyp.tura@unibs.it (P.T.); vera.radici@unibs.it (V.R.); michele.malagola@unibs.it (M.M.); 2 Hematology Division, ASST Spedali Civili Brescia, 25123 Brescia, Italy; 3 Aging Research Center, Department of Neurobiology, Care Sciences and Society, Karolinska Institutet, 17177 Stockholm, Sweden; 4 Adult Bone Marrow Transplant Unit, ASST Spedali Civili of Brescia, Department of Clinical and Experimental Sciences, University of Brescia, 25123 Brescia, Italy; 5 Laboratorio Centrale di Analisi Chimico Cliniche, ASST Spedali Civili di Brescia, 25123 Brescia, Italy; 6 Unit of Bone Marrow Transplantation and Cellular Therapies, Division of Hematology, Fondazione Istituto di Ricovero e Cura a Carattere Scientifico Policlinico San Matteo, 27100 Pavia, Italy; n.polverelli@smatteo.pv.it

## Error in Table

In the original publication [1], there was a mistake in Table 4 as published. Specifically, some numerical values were incorrectly reported as they were inadvertently taken from a different sub-analysis rather than the intended dataset.

The corrected Table 4 appears below. The authors apologize for any inconvenience caused and state that the scientific conclusions are unaffected. This correction was approved by the Academic Editor. The original publication has also been updated.

## Figures and Tables

**Table 4:** Distribution of patient characteristics across the three risk classes (frail, prefrail, and fit) of the simplified geriatric score-4 (SGS-4).

Characteristics	Total *N* = 99	Frail *N* = 13	Prefrail *N* = 62	Fit *N* = 24	*p*-Value
Age, years (range)	63.0 (56.0, 66.0)	64.0 (56.0, 68.0)	62.0 (57.0, 66.0)	63.0 (56.0, 65.0)	>0.9
Diagnosis, *n* (%)					
AL	41 (41.4)	10 (76.9)	23 (37.1)	8 (33.3)	0.10
MDS	11 (11.1)	2 (15.4)	6 (9.7)	3 (12.5)	
Lymphoma	14 (14.1)	0 (0)	11 (17.7)	3 (12.5)	
MPN	1 (1.0)	0 (0)	0 (0)	1 (4.2)	
MM	5 (5.1)	0 (0)	5 (8.1)	0 (0)	
Other	27 (27.3)	1 (7.7)	17 (27.4)	9 (37.5)	
DRI, *n* (%)					
1	2 (2.0)	0 (0)	1 (1.6)	1 (4.2)	0.08
2	70 (70.7)	7 (53.8)	50 (80.7)	13 (54.2)	
3	24 (24.3)	6 (46.2)	10 (16.1)	8 (33.2)	
4	2 (2.0)	0 (0)	1 (1.6)	1 (4.2)	
missing	1 (1)	0	0	1 (4.2)	
KPS, *n* (%)					
90–100	92 (92.9)	11 (84.6)	57 (91.9)	24 (100)	0.6
70–80	7 (7.1)	2 (15.4)	5 (8.1)	0 (0)	
HCT-CI, *n* (%)					
0	21 (21.2)	1 (7.7)	12 (19.4)	8 (33.3)	0.13
1–2	28 (28.3)	3 (23.1)	16 (25.8)	9 (37.5)	
≥3	50 (50.5)	9 (69.2)	34 (54.8)	7 (29.2)	
Disease status, *n* (%)					
1st CR	33 (33.3)	6 (46.2)	20 (32.3)	7 (29.2)	0.6
2nd or subsequent CR	66 (66.7)	7 (53.8)	42 (67.7)	17 (70.8)	
Line of Therapy, *n* (%)					
1	48 (49.5)	8 (61.5)	28 (45.2)	12 (52.0)	0.5
≥2	50 (50.5)	5 (38.5)	34 (54.8)	11 (48.0)	
Missing	1 (1.0)	0	0	1	
Stem cell source, *n* (%)					
PBSC	91(92.0)	12 (92.3)	56 (90.3)	23 (95.8)	0.9
BM	8 (8.0)	1 (7.7)	6 (9.7)	1 (4.2)	
Conditioning regimen, *n* (%)					
MAC	40 (40.4)	4 (30.7)	25 (40.3)	11(45.8)	0.7
RIC	59 (59.6)	9 (69.3)	37 (59.7)	13 (54.2)	
TCI score, *n*	2.5	2.8	2.7	3.0	0.4
Donor age, years (range)	37 (28–55)	40 (28–45)	36 (28–47)	38 (28–55)	0.9
Donor, *n* (%)					
Related	23 (23.2)	3 (23.1)	13 (21.0)	7 (29.2)	0.9
Unrelated	44 (44.5)	6 (46.1)	27 (43.5)	11 (45.8)	
Haploidentical	32 (32.3)	4 (30.8)	22 (35.5)	6 (25.0)	
Time to allo-SCT, months (range)	11.3 (5.7–54.9)	6.3 (5.7–10.2)	12.4 (7.1–29.0)	19.5 (6.7–54.9)	0.03
aGVHD	48 (48.5)	4 (30.8)	29 (46.8)	15 (62.5)	0.17
cGVHD	16 (16.2)	0 (0.0)	10 (16.1)	6 (25.0)	0.12

Table legend: *n*, number; AL—acute leukemia; MDS—myelodysplastic syndrome; MPN—Myeloproliferative Neoplasms; Multiple Myeloma—MM; DRI—Disease Risk Index; KPS—Karnofsky Performance Status; HCT-CI—Hematopoietic Cell Transplantation–Comorbidity Index; CR—complete remission; PBSCs—peripheral blood stem cells; BM—bone marrow; MAC—myeloablative conditioning; RIC—reduced-intensity conditioning; TCI—transplant conditioning intensity; allo-SCT—allogeneic stem cell transplantation; aGVHD—acute graft-versus-host disease; cGVHD—chronic graft-versus-host disease.
