# Supplementary material for: Development of a Simplified Geriatric Score-4 (SGS-4) to Predict Outcomes After Allogeneic Hematopoietic Stem Cell Transplantation in Patients Aged over 50
Source: Cancers (Basel). 2025 Oct 10;17(20):3278. doi: 10.3390/cancers17203278 (PMC12563475; doi:10.3390/cancers17203278)
Supplement: Supplementary file 1 [file cancers-17-03278-s001.zip › cancers-3896485-supplementary.pdf]

Supplementary

Table S1. Cut-off points of GCA domains.

ADL, points	<6 dependent
IAD, points	<8 dependent
G8, points	≤13 frail
VES 13, points	≤3 fraiL
CSF, points	≥3 unfit
Gait speed test, m/s	<1.1 unfit
Chair stand test, times	<14 unfit
Hand grip test, Kg	<26
Mini-Cog, points	≥3 cognitive impairment
MMSE, points	≤24 cognitive impairment
HCT-CI, points	1-2 intermediate comorbidity, ≥3 high comorbidity
FIL Score, points	considered FIT patients only those with ADL score of 6, IADL score of 8, no grade 3 – 4 comorbidities and fewer than five grade 2 comorbidities at CIRS score
